# Sarcopenia and Mortality in Critically Ill COVID-19 Patients

**DOI:** 10.3390/life14010024

**Published:** 2023-12-22

**Authors:** Fatima Al Zahra Yakti, Lana Abusalah, Vijay Ganji

**Affiliations:** 1 Human Nutrition Department, College of Health Sciences, QU Health, Qatar University, Doha P.O. Box 2713, Qatar; fy1702033@student.qu.edu.qa (F.A.Z.Y.); lana.b.abusalah@gmail.com (L.A.); 2 Department of Nutrition and Dietetics, School of Health and Human Sciences, Indiana University Indianapolis, 1050 Wishard Blvd., Indianapolis, IN 46202, USA

**Keywords:** sarcopenia, muscle mass, COVID-19, coronavirus, SARS-CoV-2, mortality, elderly

## Abstract

COVID-19 can manifest as either asymptomatic or progressing to a severe phase in some patients, which may require hospitalization. These patients may experience dyspnea and hypoxia, leading to the development of acute respiratory distress syndrome. Studies have reported an increased risk of severe sarcopenia in COVID-19 patients during and after recovery. This narrative review aimed to summarize and synthesize available studies on the association between sarcopenia and mortality in critically ill COVID-19 patients. A total of 22 studies conducted on hospitalized COVID-19 patients were included in this review. Of those, 17 studies reported a direct association, while 5 studies showed no association between sarcopenia and mortality in severe COVID-19 patients. It is important to maintain muscle quality and quantity in defense against COVID-19. The measurement of lean muscle mass should be included in the risk assessment of severely ill COVID-19 patients as part of the therapy plan.

## 1. Introduction

In 2019, a novel coronavirus disease (COVID-19), or severe acute respiratory syndrome-associated coronavirus (SARS-CoV), was first detected in China [1,2]. According to the WHO, as of December 2022, 645 million cases and 6.6 million deaths have been reported [3]. COVID-19 can be transmitted either by direct contact from person to person via respiratory airborne droplets and aerosols originating from sneezing, coughing, speaking, or by indirect contact through touching a contaminated object and then coming into coming contact with the eyes, mouth, or nose, which directly affect the respiratory system [1,2]. The complications and symptoms of COVID-19 vary among patients. The severity of the disease increases with age, the presence of comorbidities, tobacco use, immunity disorders, and obesity [4]. Most of COVID-19 patients are asymptomatic, and some may have mild symptoms such as fever, dry cough, headache, tachypnea, fatigue, sore throat, nausea, vomiting, myalgia, diarrhea, dysgeusia, or anosmia [5,6]. In some cases, COVID-19 symptoms may progress to a severe phase that requires hospitalization. These patients may suffer from dyspnea and hypoxia, leading to the development of acute respiratory distress syndrome (ARDS) [5,6]. Most post-COVID-19 patients suffer from persistent complications for several months, which may affect their mental, cognitive, and physical function [7].

Generally, sarcopenia is considered an independent risk factor for various health conditions [8]. Recently, the association between COVID-19 and sarcopenia has gained much interest. Various studies have reported that COVID-19 may lead to the development of severe sarcopenia during and after the post-recovery phase [9]. Sarcopenia is an age-related unintentional progressive loss of skeletal muscle mass and strength [10,11]. Sarcopenia can be chronic and acute. According to the European Working Group on Sarcopenia in Older People EWGSOP2, both low muscle mass and low muscle function (strength or performance) are recommended for the diagnosis of sarcopenia [12]. In comparison, acute sarcopenia is associated with a loss of physical function along with the loss of skeletal muscle mass and strength [8]. Generally, it emerges within 6 months, which is usually triggered by an acute stressor [13]. In this review, we have included all sarcopenic studies without delineating them.

In COVID-19 patients, the prevalence of sarcopenia was reported to be 48% [11]. The risk of sarcopenia and follow-up mortality is higher in hospitalized COVID-19 patients. Also, patients with COVID and sarcopenia have an extended length of hospital stay, higher admission to the Intensive Care Unit (ICU), and increased severity of disease [11]. Also, diet and nutritional status may play a significant role in COVID-19 severity [14]. To our knowledge, there was no review conducted on the relationship between sarcopenia and mortality in severely ill COVID-19 patients. This review aims to summarize and analyze the studies on the association of COVID-19 with sarcopenia on mortality in critically ill patients.

## 2. Literature Strategy

The present review is based on retrospective and prospective cohort studies, as well as cross-sectional studies. In these studies, sarcopenia was diagnosed using the measures of low muscle mass, low muscle density, and decreased muscle strength. Mortality indicators were 30-day mortality, ICU mortality, in-hospital mortality, and all-cause mortality. Commentaries and editorials were not included in this review. We conducted a comprehensive search of PubMed, Scopus, ProQuest, Google Scholar, Cochrane, and Embase databases using keywords such as “low muscle mass” OR “poor muscle strength” OR “muscle loss” OR “sarcopenia” OR “physical performance” AND “coronavirus” OR “COVID-19” OR “SARS-CoV-2” AND “mortality” OR “death”. The literature search time range was from January 2020 to December 2022. Two independent reviewers identified and selected 22 eligible articles out of 39 initially retrieved using the search strategy, as the excluded articles did not meet the inclusion criteria or were irrelevant to the topic of interest.

## 3. Potential Causes of Sarcopenia

Various risk factors can contribute to the development of sarcopenia. The natural aging process is considered a primary cause of sarcopenia [12,15], with individuals losing an average of 3 to 8% of muscle mass every year between the ages of 30 and 60 years old [16]. As a result, this is a serious and debilitating condition in older adults [17]. However, recent evidence indicates that sarcopenia may occur at any age [18]. Secondary sarcopenia may occur when risk factors such as inadequate energy intake, decreased physical activity, low-grade age-related inflammation, and disease-related systematic inflammation are present [19]. Additional factors that are responsible are malabsorption, endocrine disorders, gut microbiota dysbiosis, organ failure, muscle atrophy, insulin resistance, obesity, and the use of pharmacological drugs [17]. All these risk factors lead to elevated oxidative stress, autophagy, and decreased expression of growth factors in skeletal muscles [20]. Additionally, aging-related alterations in the structure and function of the neuromuscular junction can exacerbate sarcopenia [20].

Recent studies related to COVID-19 have shown that patients who are admitted to the ICU experience a significant loss of mass and strength of skeletal muscle [21]. A study by de Andrade-Junior et al. found that ICU bedridden patients with severe COVID-19 had a 30% reduction in the rectus femoris cross-sectional area, and after 10 days, they had a 20% reduction in the anterior section of quadriceps muscle thickness [22]. Similarly, a cohort study of 139 COVID-19 patients showed that 16% of patients were sarcopenic, and 4% had severe sarcopenia 3 months after discharge [9]. Mayer et al. reported an 18.5% reduction in the femoris rectus muscle of COVID-19 patients between the 1st and 7th of ICU admission [23]. Additionally, it was observed that 26% of patients who had not previously had sarcopenia developed sarcopenia after COVID-19 infection [24]. Because older individuals are at a higher risk for sarcopenia and are more susceptible to COVID-19 infection, they may experience a worsened sarcopenia syndrome after contracting the virus. This can result in a two-time prolonged hospital stay and an eight times higher mortality rate than non-sarcopenic COVID-19 patients [21]. Therefore, middle-aged and older adults (≥40 years old) were included in the literature search for this review. We excluded younger people because they are less susceptible to sarcopenia and less likely to experience complications associated with COVID-19.

## 4. Effect of COVID-19 on Muscles

It is becoming increasingly evident that COVID-19 can impair several organs [25,26]. It has been shown that ≈70% of patients post-COVID-19 have an impairment in one or more organs, such as intercostal muscles, lungs, bone marrow, lymphoid tissue, liver, blood vessels, and joints [25]. This impairment has been linked to an increase in serum creatine kinase and the expression of pro-inflammatory cytokines such as TNF-α, IL-1, IL-6, and IL-1β in COVID-19 patients, which significantly alters the synthesis of muscle protein [9,22]. Moreover, COVID-19 has been recognized as a major cause of malnutrition, which can negatively affect muscle mass and strength [9].

A study of post-COVID-19 patients showed that the maximal contraction for the quadriceps was 18.9 kg and for the biceps, it was 15 kg, which was estimated to be around 69% and 54% of the predicted normal value, respectively [25,27]. Furthermore, the function of these large muscle groups was impaired as well [27]. Bedrest and low physical activity are linked with declines in muscle mass and strength [28], which is usually associated with caloric insufficiency and can also lead to increased obesity and inflammation.

Among COVID-19 patients, it was observed that 10 days, post-ICU, there was a 30% loss in the rectus femoris cross-sectional area and around 20% lower thickness of the quadriceps muscle [22]. The sarcopenic respiratory disability can explain one of the main negative impacts of COVID-19 in older persons, which can increase the risk of severity and mortality of the COVID-19 infection [29]. Not surprisingly, it has been shown that COVID-19 sarcopenic patients need a twice-long hospital stay and have an eight times higher mortality rate compared to non-sarcopenic subjects [30]. All the above effects may contribute either directly or indirectly to the development of sarcopenia in several ways, from nutritional and caloric insufficiency to physical inactivity, leading to obesity and inflammation. In addition to the potential direct effect of viral infection, it produces cytokines and pro-inflammatory signaling molecules that lead to pathological effects in the skeletal muscle tissue.

## 5. Sarcopenia on Mortality in Severely Ill COVID-19 Patients

**Figure 1:**
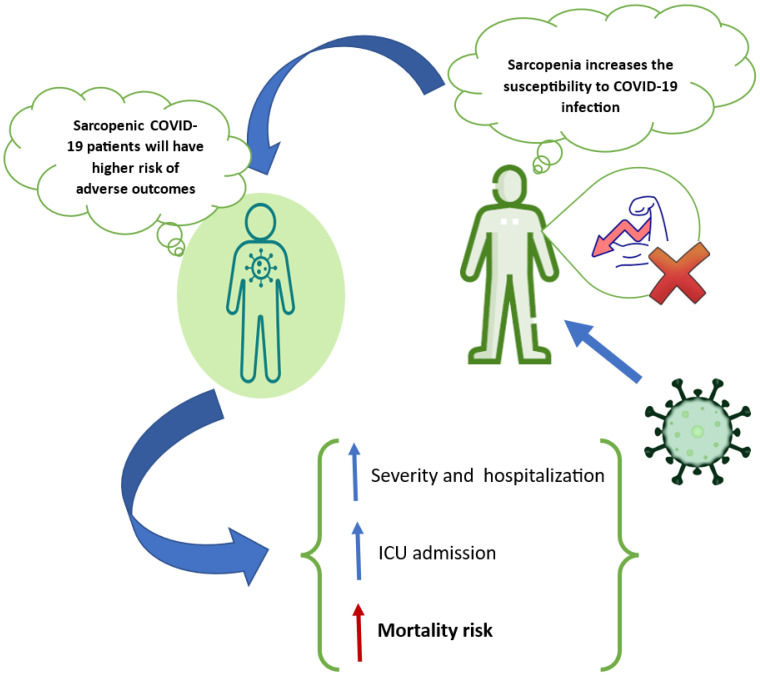
The impact of sarcopenia on mortality in COVID-19 patients.

The effect of COVID-19 disease on muscle status in patients has been the center of attention of various investigations. However, few studies addressed the effect of sarcopenia on the mortality rate among COVID-19 patients. A total of 22 studies were included in this review [30,31,32,33,34,35,36,37,38,39,40,41,42,43,44,45,46,47,48,49,50,51]. The summary of studies on the effect of sarcopenia on mortality in severe COVID-19 patients is displayed in Table 1. Data from 17 studies reported that sarcopenia was associated with mortality in severe COVID-19 patients. Most of these studies were retrospective, while two were prospective and cross-sectional. They were conducted on hospitalized COVID-19 patients between the median age of 48 and 74 years old. The studies used various tools such as computed tomography (CT) scan image and strength, assistance, rising from a chair, climbing stairs, and falls questionnaire to assess sarcopenia. The impact of sarcopenia on mortality in COVID-19 patients is shown in Figure 1.

## 6. Sarcopenia on Mortality in Critically Ill Patients

Multiple organ dysfunction was common in critically ill patients receiving intensive care, which raised the mortality risk. Thanks to significant advancements in medical care, mortality rates have progressively declined over time, particularly for oncology and hematological patients [52] several factors, including malnutrition, sepsis, immobilization, and multiple organ dysfunction syndrome [53,54,55,56,57,58]. Consequently, stratifying the risk of death and predicting mortality is crucial. According to a growing number of research, sarcopenia is typically a problem for critically sick patients because of factors like dietary status, inflammation, the presence of other diseases, and inactivity [56,59]. In intensive care units, sarcopenia is thought to affect between 30 and 70 percent of patients [57,58,60,61]. Sarcopenia has been linked to negative clinical outcomes in older persons in the community, nursing homes, or ICU, including falls, fractures, poor quality of life, death, and cognitive dysfunction [59,60,61,62,63,64,65]. In a recent meta-analysis, Xia and colleagues found that injured individuals with sarcopenia have a two-fold higher risk of mortality versus those without sarcopenia [63,66]. Also, Moisey et al. concluded that critically ill elderly patients with identified sarcopenia had significantly higher mortality incidence compared to non-sarcopenic patients [57,60]. Similar findings have been seen in a meta-analysis done by Xiao and colleagues deduced that regardless of short- or long-term mortality, critically ill individuals with sarcopenia have a 2.28-fold lower chance of survival compared to non–sarcopenic individuals [64,67]. In addition to these, critically sick patients frequently experience complications from deteriorating conditions, including severe inflammation, malnutrition, starvation, and multiple organ failure, which worsen patients’ illnesses in a vicious cycle due to the interplay of sarcopenia [64,65,66,67,68]. Additionally, sarcopenia-related critical illness may make it more likely that intensive care treatments, such as the use of multiple medications, bed rest, sedation, instrumentation, and mechanical breathing, would have unfavorable side effects [66]. The risk of fatality will rise in sarcopenic patients who become severely ill due to these various conditions [67].

Schiaffino et al. [31] conducted a study aimed to investigate the potential impact of muscle status derived from axial chest CT scans on forecasting the clinical outcome of COVID-19 patients, such as in-hospital mortality. The study assessed three muscles, including skeletal muscle mass, paravertebral muscle, and dorsal muscle at the level of T5 and T12, in 552 hospitalized COVID-19 patients. The study concluded that at level T5, the pectoralis muscle area (PMA) was significantly associated with death (*p* = 0.001). The findings of this study were consistent with the conclusions of Erkan et al. [32], who studied the effect of sarcopenia on in-hospital mortality independently of other demographic factors. They found that Sarcopenia was significantly associated with the mortality group compared to the non-mortality group (*p* < 0.001). Sarcopenia was measured at the level of T12, and they conducted a ROC curve analysis to obtain their sarcopenia cutoff values (34.06 cm^2^/m^2^ in men and 29.36 cm^2^/m^2^ in women). Kim et al. [30] and Erdöl et al. [33] also found consistent results. Both studies assessed skeletal muscle at a level of T12. Erdöl et al. [33] assessed the association between in-hospital mortality and three skeletal muscles (erector spinal muscle, pectoralis muscle, and total skeletal muscle) and found that the mortality rate was highest in the third tertile of SMcsa index (*p* < 0.001). However, these results were not independent of other factors, as COVID-19 patients had risk factors for CVD. On the other hand, Kim et al. [30] found that the association between sarcopenia and mortality rate was independently significant (*p* < 0.004). They observed that the incidence of mortality was higher among sarcopenic COVID-19 patients than those without sarcopenia. The systematic inflammation associated with COVID-19 is the reason for the direct association between sarcopenia and mortality [30].

Two studies done in Turkey by Ufuk et al. [34] and Hocaoglu et al. [35] investigated the link between the decrease in pectoralis muscle and the occurrence of death in patients with COVID-19. They predicted sarcopenia diagnostic cutoff values based on different estimations. Ufuk et al. [34] estimated and divided the pectoralis muscle index into tertile based on gender-specific pectoralis muscle index (PMI). The smallest tertile was categorized as having a low PMI of ≤12.73 cm^2^/m^2^ for men and ≤9 cm^2^/m^2^ for women. According to their diagnostic criteria for sarcopenia, they deduced that the low PMI tertile was associated with a risk of death in patients during observations for 1-month follow-up (*p* = 0.019) [34]. Hocaoglu et al. [35] used ROC analysis to estimate pectoralis muscle density cutoff values (≤15.9 in women; ≤34.1 in men). They found that the mortality rate was 4.446 times higher in women and 4.3 times higher in men with severe COVID-19 disease with low PD levels in patients aged ≥65 years old compared to those less than 65 years. However, some studies reported mixed findings. Polat et al. [36] and Attaway et al. [37] found an inverse association between low pectoralis muscle and pectoralis muscle index, which were measured at the level of T12 and L2, with in-hospital mortality (*p* ˂ 0.01). However, Attaway et al. [37] reported contradictory results in their cohort, where they showed no difference between loss in erector spinal muscle and mortality, but it was significantly related to ICU admission. A similar observation was found by Polat et al. [36], where they found no difference between psoas density and psoas CSA and mortality and ICU admission.

Several studies have reported that survivors of COVID-19 had higher values for various parameters of pectoralis muscle (PM) compared to non-survivors. A retrospective cohort study by Surov et al. [38] found that survivors had higher values for all the parameters (area, index, gauge, and density) of the PM versus non-survivors (*p* < 0.01). Similarly, Poros et al. [39] conducted a retrospective study on 74 patients with severe COVID-19 patients who were aged 66 years old and found that deceased patients had a lower CSA and PMA in both genders (*p* ˂ 0.001). However, this study included a small number of patients compared to Surov et al.’s study [38]. Another cohort study by Hosch et al. [40] followed hospitalized COVID-19 patients for 24 months and 12 days and found that sarcopenia was significantly associated with disease severity and mortality rate (*p* < 0.0001).

Assessment of skeletal muscle loss by CT image slice at L3 for the thorax, abdomen, and pelvis has been conducted by several studies, including Nobel et al. [41] and McGovern et al. [42]. Nobel et al. [41] aimed to determine the link between body composition risk factors such as skeletal muscle loss and 30-day mortality in COVID-19 inpatients presented with gastrointestinal (GI) symptoms. Their findings demonstrated that patients without GI symptoms who died had less skeletal mass index (SMI) (*p* = 0.010) compared to patients with GI symptoms. McGovern et al. [42] found that sarcopenia was significantly associated with a high incidence of 30-day mortality in the presence of obesity (*p* < 0.05).

In addition, two other studies assessed sarcopenia using the SARC-F questionnaire on hospitalized elderly COVID-19 patients. Riesgo et al. [43] predicted sarcopenia in hospitalized patients (*n* = 337) based on the SARC-F questionnaire (value of >4 as a prediction of sarcopenia) and found that it was independently associated with mortality (*p* = 0.04). Similarly, de Silva et al. [44] assessed and predicted the risk of being sarcopenic in elderly patients by using the SARC-F questionnaire and found that patients whose diet had a higher risk of being sarcopenic compared to patients who were discharged. Piotrowicz et al. [45] conducted an observational prospective study on 163 hospitalized severe COVID-19 male patients. The authors assessed sarcopenia according to the EWGSOP2 guidelines and followed the SARC-F questionnaire with a value of >4. Results showed a significant mortality risk of 441% greater among patients with probable sarcopenia. Finally, Damanti et al. [46], in an observational study, found that muscle mass was related to lowered hospital mortality (*p* = 0.02) and muscle density was inversely associated with length of hospitalization (*p* = 0.02) and in-hospital mortality (*p* = 0.046) in 81 inpatients.

Out of 22 studies we reviewed, five studies found no relation between sarcopenia and mortality risk in COVID-19 patients. A retrospective investigation by Kang et al. [47] aimed at studying the relationship between muscle loss and 4-month mortality in Korean patients with COVID-19. They showed that after following patients for 4 months, there was no association between sarcopenia and mortality risk or any of the clinical outcome measurements [47]. The limitations of this study were a small sample size and the possibility of selection bias [47]. Comparable findings were also found in two retrospective cohort studies [48,49]. Moctezuma-Velázquez et al. assessed SMI at a level of T12 based on analysis of a transverse CT scan image [48]. They found no association between low SMI and in-hospital mortality, ICU admission, and invasive mechanical ventilation for both genders. The authors concluded that SMI does not have a predictive role in adverse clinical outcomes, and acute COVID-19 infection is more strongly linked to mortality [48]. Kardas et al. [49] found that PMI and PMA were not significantly associated with 30-day mortality and length of hospital stay. The small sample size is a limitation of this study. Antonarelli et al. [50] examined the muscle status of 112 elderly COVID-19 patients with a median age of 60.5 years who were hospitalized. They calculated PMA and PMI at the level of T4 and found that sarcopenia was associated with longer ICU admission but not with inpatient mortality. Graziano et al. [51] findings showed no significant difference between sarcopenia and clinical outcomes such as 30-day mortality and ICU admission.

## 7. Conclusions

Based on the current overwhelming evidence, sarcopenia is associated with ICU admission, increased hospital stays, and increased risk of mortality among COVID-19 patients. This can occur due to a variety of factors, including calorie insufficiency or malnutrition for maintaining muscle mass [68], obesity, and inflammation, as well as direct effects of the viral infection on skeletal muscle tissue. Because COVID-19 patients with sarcopenia had a higher risk of developing poor clinical outcomes, they should be prioritized to receive the COVID-19 vaccine. This review highlights the importance of maintenance of muscle quality and quantity in the defense against COVID-19 infection. This is not limited to COVID-19, but it can also be applied to other infectious diseases that can threaten the lives of older individuals. Therefore, periodic tests of body composition, especially among the middle-aged and elderly populations, should be incorporated as part of the therapy plan for critically ill patients.

Studies on the relationship between sarcopenia and mortality from COVID-19 infection used different criteria and definitions for diagnosing sarcopenia, which may have contributed to some heterogeneity in the findings. The association between sarcopenia and mortality in children has not been investigated. It is possible that younger patients may be less susceptible to muscle loss and sarcopenia or less likely to develop complications associated with COVID-19 infection. Therefore, these findings cannot be applied to younger populations. Most of the studies on sarcopenia and mortality in COVID-19 patients are retrospective in nature; therefore, interventional trials are needed.

## Figures and Tables

**Table 1:** Summary of the studies on the association between sarcopenia and mortality in severe COVID-19 patients ^1^.

Authors and Country	Study Design	Population & Sample Size, n	Median Age, y	Sarcopenia Assessment	Sarcopenia Diagnostic Criteria	Mortality Definition	Follow Up, mo/d	Main Findings-Mortality	Other Findings
Studies on the direct association between sarcopenia and mortality									
Kim et al. [30] South Korea	Retrospective cohort study	Hospitalized COVID-19 patients, *n* = 121 (44 men; 77 women)	62	Axial chest CT scan at the level of T12 (SMI)	SMI (men, ≤24 cm^2^/m^2^; women, ≤20 cm^2^/m^2^)	Mortality	4 mo	Sarcopenia was associated with mortality but not independently (*p* = 0.004)	Sarcopenia was independently associated with a longer time to discharge (*p* < 0.001)
Schiaffino et al. [31] Italy	Multicenter Retrospective observational study	Hospitalized COVID-19 patients, *n* = 552 (364 men; 188 women)	65	Axial Chest CT image, all muscles at T5 and T12 level (DMI, PMA, PMI, SMM)	-	In-hospital mortality	2 mo, 21 d	Low PMA was associated with death (*p* = 0.001)	Low PMA was associated with ICU admission (*p* < 0.001)
Erkan et al. [32] Turkey	Retrospective study	Hospitalized COVID-19 patients, *n* = 302 (146 men; 156 women)	56.7 to 69.7	Axial Chest CT scan at a level of T12 (SMA)	-SMI (men, 34.1 cm^2^/m^2^; women, 29.4 cm^2^/m^2^) -Univariate and multivariate analysis	In-hospital mortality	-	Sarcopenia was associated independently with mortality (*p* < 0.001)	Sarcopenia was associated with hospitalization and ICU admission (*p* < 0.001)
Erdöl et al. [33] Turkey	Retrospective cohort study	Hospitalized COVID-19 patients with >1 CVD risk factor, *n* = 232 (117 men; 115 women)	51	Chest radiographs and axial CT scan at a level of T12 (CSA, SM, ESM, PM)	-SM-CSA (<21.7 cm^2^) -ESM (<11.4 cm^2^/m^2^) -PM (<10.3 cm^2^/m^2^)	-In-hospital mortality -All-cause mortality	1–48 mo	Low SM-CSA (Tertile 3) had the highest mortality rate (*p* < 0.001)	Diabetes and hypertension in addition to sarcopenia were associated with in-hospital mortality
Ufuk et al. [34] Turkey	Retrospective study	Hospitalized COVID-19 patients, *n* = 130 (76 men; 54 women)	48	Axial chest CT image (CSA, PMA, PMI)	PMI (men, ≤12.7 cm^2^/m^2^; women, ≤9 cm^2^/m^2^)	Death during follow-up	1	Low PMI associated with death (*p* = 0.019)	Low PMI was associated with longer hospital stays (*p* = 0.01)
Hocaoglu et al. [35] Turkey	Retrospective study	Hospitalized COVID-19 patients, *n* = 217 (108 men; 109 women)	61	Axial Chest CT scan (PMV, PD)	PD (women, ≤15.9; men, (≤34.1)	In-hospital mortality	-	A significant association between mortality and PD of ≤15.9 in women and ≤34.1 in male (*p* = 0.001)	Low PV associated with increased severity
Polat et al. [36] Turkey	Retrospective	Hospitalized COVID-19, male patients, *n* = 130	74	Single-axial chest CT image at level of L2 (Psoas CSA, Psoas density, Psoas MI)	-	In-hospital mortality	2	-Low PMI is significantly associated with mortality (*p* = 0.001). -No significant between psoas density and psoas CSA & mortality	No significant association between Psoas CSA, Psoas density, PMI, and ICU admission
Attaway et al. [37] USA	Retrospective cohort study	Hospitalized COVID-19 patients, *n* = 95 (50 men; 45 women)	63.3	Axial chest CT scan, PM above the aortic arch & ESM at a level of T12 (CSA, PM, ESM)	-PM (<29 cm^2^). -ESM (<35.2 cm^2^)	In-hospital Mortality	10 mo	Loss of PM was associated with mortality (*p* = 0.006), while a loss in ESM was not (*p* = 0.089)	Loss in PM was associated with ICU admission (*p* = 0.006)
Surov et al. [38] Turkey	Retrospective cohort study	Hospitalized COVID-19 patients, *n* = 1138 (591 men; 547 women)	54.5	Axial thoracic CT at a level of T4 (PMA, PMI, PMD, and PMG)	PMA, PMI, PMD, and PMG	30-d mortality	-	PM parameters (PMA, PMI, PMD, and PMG) were associated with mortality (*p* < 0.001)	Lower parameters of the PM were associated with unfavorable outcomes (*p* < 0.01)
Poros et al. [39] Germany	Retrospective	Hospitalized COVID-19 patients, *n* = 74 (60 men; 14 women)	66	Thoracic CT scan at the level of T5 (PMA, CSA)	-	In-hospital mortality	1	Died patients had lower muscle CSA and PMA. (*p* < 0.001)	-≥65 y had lower thoracic SMM compared to ≤65 y -Men had greater thoracic SMM with less ventilation and ICU need than women
Hosch et al. [40] Germany	Retrospective cohort study	Hospitalized COVID-19 patients, *n* = 918 (564 men; 354 women)	78	CT thorax scan	-	In-hospital mortality	24 mo, 12 d	Sarcopenia had a significant association with mortality (*p* < 0.0001)	-Sarcopenia had a significant association with severity (*p* < 0.0001). -Cardiac marker was only associated with severity (*p* < 0.0001)
Nobel et al. [41]	Retrospective cohort study	Hospitalized COVID-19 patients, *n* = 190 (105 men; 85 women)	64	Abdomen axial CT slice at the L3 vertebral level (SMI)	-	30-d mortality	1 mo, 5 d	Patients who died had less SMI (*p* = 0.01)	Patients who died had greater IMAT area, VAT area, and SAT (*p* = 0.049)
McGovern et al. [42] UK	Retrospective cohort study	Hospitalized COVID-19 patients, *n* = 63 (30 men; 33 women)	67% had the age of >70	CT image at L3 for thorax, abdomen, and pelvis (SMI)	Men: BMI < 25 kg/m^2^ & SMI < 43 cm^2^/m^2^, or BMI ≥ 25 & SMI < 53 cm^2^/m^2^. Women: BMI < 25 & SMI < 41 cm^2^/m^2^, or BMI ≥ 25 & SMI < 41 cm^2^/m^2^	30-d mortality	1 mo, 13 d	Low SMI was associated with 30 d mortality (*p* < 0.05)	High VFA was associated with 30 d mortality (*p* < 0.05)
Riesgo et al. [43] Spain	Cross-sectional study	Hospitalized COVID-19 patients, *n* = 337 (167 men; 170 women)	86.1	SARC-F questionnaire	ASRC-F score of ≥4 is predictive of sarcopenia	Mortality	5 mo	Only SARC-F score ≥4 was independently associated with mortality (*p* = 0.01)	Inpatients who died, their age was higher (*p* = 0.01), while albumin was lower (*p* = 0.01)
de Silva et al. [44] Brazil	Historical cohort study	Hospitalized COVID-19 patients, *n* = 222 (125 men; 97 women)	62.8	SARC-F questionnaire	>4 as predictive of sarcopenia	In-hospital mortality	9 mo	Sarcopenia was higher in dead patients than survivors (*p* < 0.001)	BMI and albumin were lower in dead patients (<0.001, *p* = 0.009, *p* < 0.001)
Piotrowicz et al. [45] Poland	Prospective, cohort study	Hospitalized COVID-19 patients, *n* = 163 (90 men; 73 women)	≥65	EWGSOP2 guidelines	≥4 points as predictive of sarcopenia	-In-hospital mortality -3-mo post-discharge	3 mo	Sarcopenia is associated with greater mortality risk by 441% (*p* = 0.01)	No significant association
Damanti et al. [46] Italy	Retrospective cohort study	Hospitalized COVID-19 patients, *n* = 81 (71 men; 10 women)	59.3	Axial chest CT scan at level of L3 (SMI, Muscle density, CSA)	SMI (women, 34.4 cm^2^/m^2^; men, 45.4 cm^2^/m^2^)	-In-hospital mortality -Mortality in ICU	2 mo, 4 d	-Muscle CSA and density were inversely associated with hospital mortality (*p* = 0.02, *p* = 0.046). -SMI was inversely associated with hospital (*p* = 0.002) and ICU (*p* = 0.008) mortality	Muscle density had an inverse association with the complications in ICU (*p* = 0.03), length of hospitalization (*p* = 0.002)
Studies on no association between sarcopenia and mortality									
Kang et al. [47] South Korea	Retrospective study	Hospitalized COVID-19 patients, *n* = 127 (67 men; 60 women)	61	Cross-sectional Chest CT image, at level of L2 (SMA, SMI)	SMI (men, <50 cm^2^/m^2^; women, <39 cm^2^/m^2^)	4-mo mortality	4 mo	Sarcopenia was not associated with 4-mo mortality.	N/A
Moctezuma- Velázquez et al. [48] Mexico	Retrospective cohort	Hospitalized COVID-19 patients, *n* = 519 (332 men; 187 women)	51	Transverse CT scan image e at the level of T12 (SMI)	SMI (men, <42.6 cm^2^/m^2^; women, <30.6 cm^2^/m^2^)	In-hospital mortality	3 mo, 12 d	No association between low SMM, SMI, SMA, and mortality	No significant association between low SMM, SMI, and SMA with invasive mechanical ventilation and ICU admission
Kardas et al. [49] Germany	Retrospective cohort study	Hospitalized COVID-19 patients, *n* = 46 (27 men; 19 women)	64.5	Axial chest CT scan at the level of T4 (PMI, PMA, SMG)	-	30-d mortality	3 mo	Sarcopenia measures were not associated with 30 d mortality (*p* > 0.05)	
Antonarelli et al. [50] Italy	Retrospective study	Hospitalized COVID-19 patients, *n* = 112 (82 men; 30 women)	60.5	Axial chest CT image at a level of T4 (PMA, PMI, PMD)	-	In-hospital mortality	9	No association between PMA and PMD with mortality	Higher PMA and PMD associated with shorter ICU stay (*p* = 0.0034), (*p* = 0.0002)
Graziano et al. [51] Italy	Prospective cohort study	Hospitalized COVID-19 patients, *n* = 195 (126 men; 69 women)	71	SECA, using tetrapolar method (SMM)	SMM/BMI ratio (men, 1.05 kg/m^2^; women, 0.71 kg/m^2^)	-In-hospital mortality -30-d mortality	1	Sarcopenia was not associated with 30-d mortality. (*p* = 0.211)	Sarcopenia was associated with a need for ventilator (*p* = 0.051), but not ICU admission nor length of stay

^1^ Abbreviations: BMI: body mass index; COVID-19: Coronavirus disease-2019; CSA: cross-sectional area; CT: computed tomography; CVD: cardiovascular diseases; ESM: erector spine muscle; EWGSOP2: European Working Group on Sarcopenia in Older People; GI: gastrointestinal; ICU: intensive care unit; IMAT: intramuscular adipose tissue; N/A: not applicable; PM: Pectoralis muscle; PMA: pectoralis muscle area; PMD: pectoralis muscle density; PMG: pectoralis muscle gauge; PMI: pectoralis muscle index; PMV: pectoralis muscle volume; PV: pectoralis muscle volume; SARC-F: Strength, Assistance, Rising from a chair, Climbing stairs, and Falls; SAT: subcutaneous adipose tissue; SECA: scale up line that calculates the vital whole body composition parameters; SM: Skeletal muscle; SM-CSA: skeletal muscle-cross sectional area; SMG: skeletal muscle gauge; SMI: skeletal muscle index; VAT: visceral adipose tissue; VFA: visceral fat area.

## Data Availability

No new data were collected. Therefore, this is not applicable.
